# Dissected Thoracic Aorta Masked as Seizures: A Case Report

**DOI:** 10.3390/jcm15031148

**Published:** 2026-02-02

**Authors:** Paweł Chochoł, Anna Witt-Majchrzak, Marcin P. Mycko

**Affiliations:** 1 Department of Neurology and Neurosurgery, Medical Division, University of Warmia and Mazury in Olsztyn, 10-719 Olsztyn, Poland; marcin.mycko@uwm.edu.pl; 2 Cardiothoracic Surgery Department, The Regional Specialist Hospital in Olsztyn, 10-561 Olsztyn, Poland; awitt@wss.olsztyn.pl

**Keywords:** aortic dissection, acute aortic syndrome, seizure, case report

## Abstract

**Background**: Aortic dissection (AoD) is a life-threatening medical emergency characterized by the separation of the layers of the aortic wall. The typical clinical presentation of AoD includes intense thoracic pain in the anterior chest or interscapular region, often described as migratory and tearing in nature. However, in rare cases, AoD can present without classic signs but with neurological symptoms, including seizures. **Case Presentation**: A 60-year-old patient experienced a sudden loss of consciousness followed by a tonic–clonic seizure and subsequently developed right-sided weakness. He had a medical history of hypertension and smoking. Although the symptoms quickly resolved, the brain imaging revealed signs of an acute ischemic stroke located in the left hemisphere. The seizures resumed, blood D-dimer levels were found to be highly elevated, and subsequent thoracic and abdominal computed tomography angiography revealed the presence of AoD, which originated at the proximal part of the ascending aorta. The patient received symptomatic treatment to alleviate his symptoms and prevent complications and was quickly transferred for surgical intervention, resulting in a favorable outcome. **Conclusions**: This case demonstrates that a tonic–clonic seizure can be the first clinical manifestation of AoD. Such atypical symptoms highlight the diverse and misleading nature of AoD presentations, underscoring the challenges in the diagnostic process. This emphasizes the need for increased clinical vigilance when treating a patient experiencing their first seizure episode.

## 1. Background

Aortic dissection (AoD) is defined as a separation of the layers composing the aortic wall and considered the most prevalent aortic pathology. Classically, the dissection plane is described between the intima and the muscular layer; but it may also involve the media or even a separation of the media from the adventitia. The typical clinical presentation of AoD is described as intense thoracic pain in the anterior chest or interscapular dorsal region, with migratory and tearing characteristics often associated with dysautonomic phenomena such as diaphoresis. Aortic dissections are classified by chronicity as hyperacute (<24 h), acute (2–7 days), subacute (8–30 days), and chronic (>30 days) [1]. AoD is the most prevalent aortic pathology, with an incidence of 2.6–3.5 cases per 100,000 inhabitants per year [2]. Morbidity and mortality rates have been estimated to be as high as 90% at 3 months without proper and timely management, with an increase rate of 1% per initial hour if untreated within the first 24 h [3]. Despite typical symptoms, it has been reported that up to 38% of cases go unnoticed in an initial evaluation, either due to additional symptomatology or the absence of classic symptoms, and in 28% of cases the diagnosis is made post-mortem, highlighting the severity and importance of timely diagnosis [4].

The objective of this paper is to provide a case report demonstrating an unusual clinical course of AoD resulting in a neurological manifestation. This rare case proves that AoD symptoms could be atypical and exemplifies the difficulty of diagnosing AoD promptly in clinical practice.

## 2. Case Presentation

A 60-year-old male was brought by ambulance to the Department of Neurology following an episode of sudden loss of consciousness accompanied by tonic–clonic seizures. This episode was followed by right-sided limb weakness and speech difficulties. His medical history included hypertension, for which he had been treated with telmisartan and hydrochlorothiazide for the last four years, as well as a history of gallstones and kidney stones. The patient reported an average blood pressure of 130/85 mmHg. He had no previous history of seizures or sudden fits and was a long-term smoker, having smoked approximately 25 cigarettes a day for at least forty years.

Upon admission, the patient was conscious but unresponsive and reacted only to strong stimuli. He exhibited paresis in the right upper limb, with slight movement in the right lower limb. The National Institutes of Health Stroke Scale (NIHSS) scored him at 19 points, while his disability status, assessed using the modified Rankin scale (mRS) was 5 points. There were no signs of dyspnea or chest pain, and heart rate and blood pressure were normal.

Within the first hour of hospitalization, the patient’s symptoms improved. Upon re-examination, he was conscious; followed instructions; and was oriented to person, place, and time. He showed no signs of meningeal inflammation, cranial nerve deficits, or limb weakness. Pathological pyramidal signs were negative, coordination was normal, and he was able to walk without assistance. His NIHSS and mRS scores improved to 0 points. The patient’s vital signs remained stable, with blood pressure fluctuating between 90/70 mmHg and 150/90 mmHg and a heart rate ranging from 54 to 72 beats per minute.

Laboratory blood tests upon admission yielded normal results, including electrolytes, hemoglobin, troponin levels, coagulation parameters, creatinine, glucose, and C-reactive protein. The serum lipid profile showed slightly elevated levels of total cholesterol (191 mg/dL) and triglycerides (152 mg/dL). A urine test indicated signs of a urinary tract infection.

**Figure 1:**
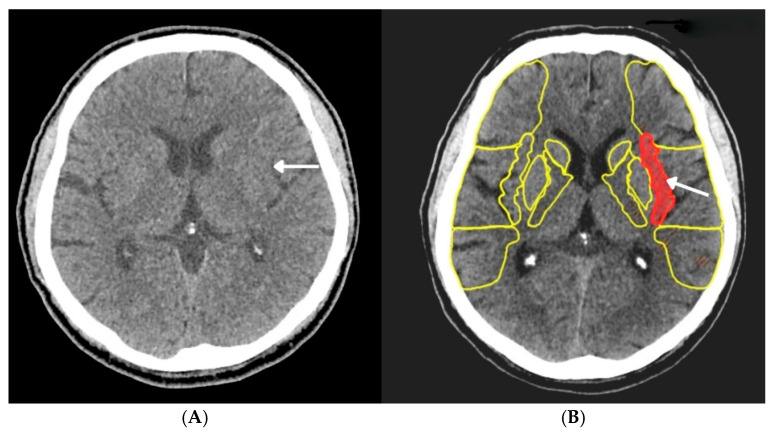
Computed tomography of the head revealing a discreet blurring of the left insular band (**A**) and left insular ribbon involvement (area marked in red) according to Alberta stroke program early CT score (ASPECTS) (**B**) (marked by arrow).

A computed tomography (CT) scan of the head revealed no signs of bleeding, but it did show discreet blurring of the insular band on the left side (Figure 1A). The Alberta Stroke Program Early CT Score (ASPECTS) was assessed by e-ASCPECTS version 12 software (Brainomix, Oxford, UK) as 9 out of 10 (Figure 1B). The ventricular system appeared normal, without displacement or dilation. An angio-CT scan (CTA) of cervical and brain arteries from the aortic arch to the skull vault demonstrated an occluded left common carotid artery (LCCA) just behind its origin, with the left internal carotid artery (LICA) poorly contrasted—likely due to retrograde filling. The right CCA and ICA were patent, as were the vertebral arteries; basilar artery; and anterior, middle, and posterior cerebral arteries, with no significant stenoses. No aneurysms were observed. This imaging did not reveal any obvious signs of the aorta dissection. 

**Figure 2:**
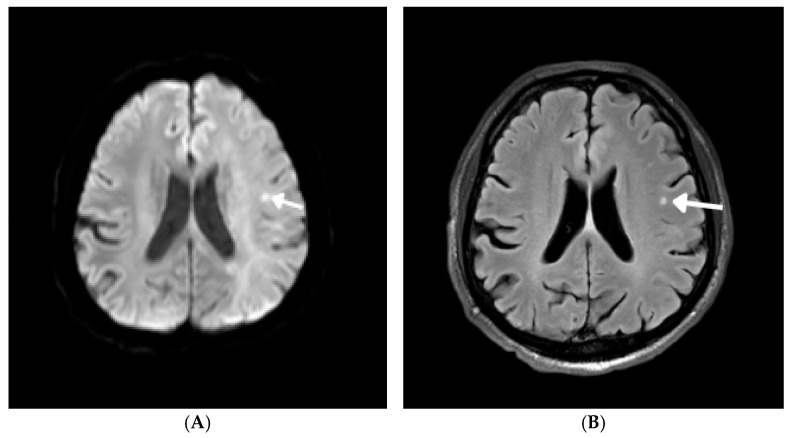
Head magnetic resonance imaging with DWI sequence (axial) (**A**) and FLAIR sequence (axial) (**B**) with abnormal hyperintense signal in the left frontal region (marked by arrow).

The head magnetic resonance imaging (MRI) revealed, in the diffusion-weighted imaging (DWI) sequence, two mildly hyperintense foci in the left hemisphere—located in the frontal and parietal regions—with diameters of up to 5 mm (Figure 2A). The parietal focus did not show signal changes in the FLAIR sequence and was hypointense on the apparent diffusion coefficient (ADC) maps, indicating an acute ischemic change. In contrast, the frontal focus was hyperintense in the FLAIR sequence and on ADC maps, suggesting it was an ischemic change in the subacute phase. Additionally, T2 and FLAIR images showed several small hyperintense foci, measuring up to 4 mm in diameter, in the subcortical white matter of both hemispheres, likely of vascular origin and occurring at different times (Figure 2B).

**Figure 3:**
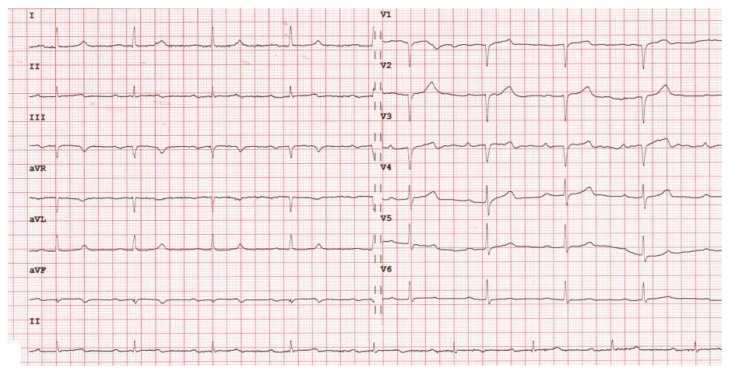
Patient’s electrocardiogram, timebase 25 mm/s.

**Figure 4:**
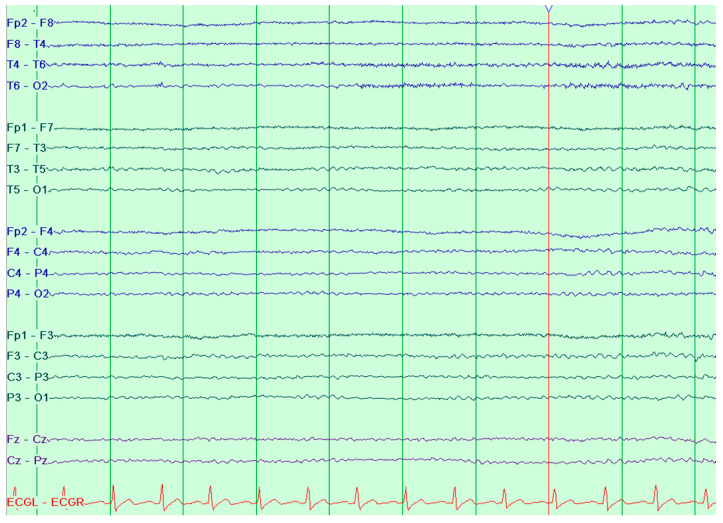
Patient’s electroencephalogram, longitudinal bipolar montage, sensitivity 10 uV, timebase 30 mm/s.

The electrocardiogram (ECG) showed a sinus rhythm with no other abnormalities (Figure 3). The electroencephalogram (EEG) was reported as normal, exhibiting alpha activity at 8 Hz with an amplitude of up to 30 µV in the posterior areas of the brain; activations did not change the recording (Figure 4). Initially, upon complete neurological recovery to NIHSS 0, the patient was administered dual antiplatelet therapy, consisting of aspirin at 75 mg and clopidogrel at 75 mg, taken orally. Additionally, atorvastatin (40 mg) was given once daily, along with prophylactic low-molecular-weight heparin (LMWH) at a dosage of 0.04 g/0.4 mL, and intravenous fluid therapy was provided. Also, hypertensive treatment was continued with amlodipine at 5 mg and telmisartan at 80 mg once daily.

On the next day of hospitalization, the patient experienced a second episode of sudden loss of consciousness accompanied by a generalized tonic–clonic seizure. For this, he was administered a bolus of 10 mg diazepam intravenously, followed by 300 mg of valproate orally. The seizures did not reoccur, and the patient regained consciousness.

**Figure 5:**
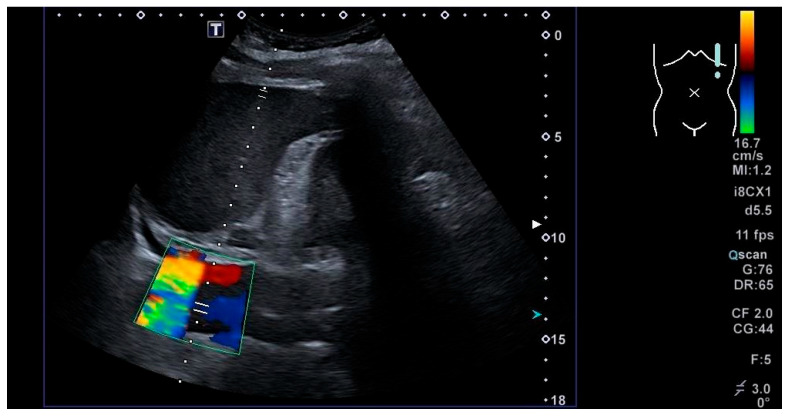
Patient’s echocardiogram.

At this time, a new set of blood tests was performed, which indicated elevated levels of CRP (112 mg/L (reference range: 0–5)) and a D-dimer level of 7.44 µg/L (reference range: 0–0.5). The echocardiogram revealed left ventricular (LV) wall hypertrophy, a significantly dilated ascending aorta measuring up to 6.5 cm with a visible dissection channel, severe aortic regurgitation, and a pericardial effusion behind the right ventricle measuring up to 1 cm. Additionally, there was impaired diastolic function, although LV contractility was unaffected (Figure 5).

**Figure 6:**
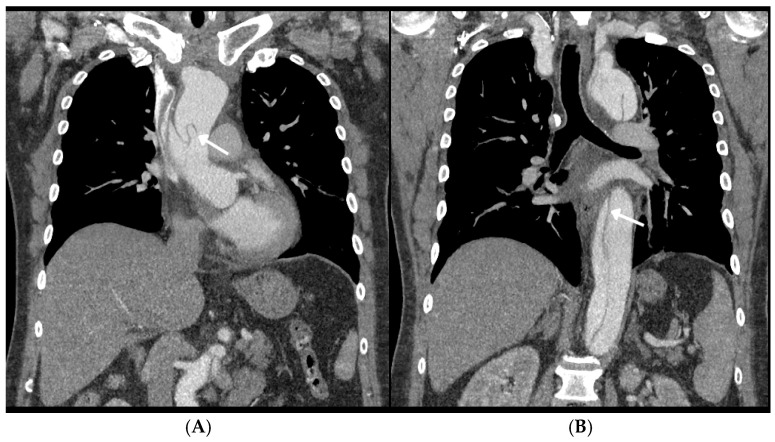
Computed tomography angiography (CTA) with the dissection of the proximal ascending (**A**) and descending (**B**) parts of the aorta (marked by arrow).

In order to investigate the vascular status of the patient, an additional thoracic and abdominal angio-CT was ordered. This clearly revealed a dilated thoracic aorta with features of dissection starting just above the aortic bulb (Figure 6A) and extending to the origin of the left renal artery (Figure 6B). The aorta dissection also involved the brachiocephalic trunk and the initial part of the right common carotid artery (RCCA), which was not visible in the previous angio-CT examination of the neck and head arteries. There were also indications of dissection in the initial section of the left common carotid artery (LCCA) and suspicion of significant stenosis in the left subclavian artery. The celiac trunk showed dissection in its initial section, but it was otherwise properly contrasted. The superior mesenteric artery exhibited signs of dissection, and there was suspected dissection in the initial section of the left renal artery, although it appeared that renal arteries were being contrasted from the same channel. Below the renal artery branching, the left aorta showed no signs of dissection. The iliac arteries (common, external, and internal) were free of dissection. The dimensions of the aorta were reported as follows: bulb—59 mm, ascending aorta—53 mm, arch—43 mm, descending aorta—44 mm, celiac trunk outlet—36 mm, renal arteries—28 mm, and before division—20 mm. Additionally, there was a trace of fluid in the pericardium. The pulmonary trunk and its lobar and segmental branches showed no obvious contrast defects typical of central pulmonary embolism, and the pulmonary trunk was not dilated. Fluid was present in the pleural cavities—up to 20 mm on the left side and up to 5 mm on the right side. In conclusion, the findings were consistent with signs of aortic dissection classified as type A Stanford/I DeBakey.

The patient was urgently consulted by a cardiac surgeon and was deemed suitable for immediate surgery. He was transferred to the Cardiothoracic Surgery Department for further management. Upon discharge from the Neurology Department, the patient was conscious with no focal symptoms. His NIHSS and mRS scores were 0, his blood pressure was 90/70 mmHg, his heart rate was stable at 80 beats per minute, and his blood oxygen saturation was above 90%.

After echocardiographic verification of the aortic valve, the patient was qualified for Bentall surgery. The operation was performed under extracorporeal circulation with hypothermia set at 30 degrees Celsius. During the procedure, a conduit was implanted along with a mechanical prosthesis of the SJM 27 mm aortic valve. In the postoperative course, the patient required circulatory support with catecholamine intravenous infusions for four days. Acute postoperative renal failure was identified, necessitating continuous renal replacement therapy. There was also an air leak in the chest tube, which required tube maintenance for up to six days after the surgery. The patient’s postoperative treatment included intravenous administration of adrenaline, dobutamine, and noradrenaline, along with intravenous fluids and furosemide. Analgesics were also provided, and perioperative prophylaxis was administered using cefazolin, later switching to ceftriaxone; LMWH was also included in the treatment regimen. Due to the presence of atrial fibrillation and second- and third-degree atrioventricular blocking identified through a long-term ECG examination, a VVIR-type pacing system was implanted. Anticoagulant therapy with warfarin was initiated later, once the risk of bleeding was reduced, with careful monitoring of the international normalized ratio (INR) to ensure that it remained stable. After 16 days post-surgery, the patient was discharged home in a good condition, with stable circulatory and respiratory functions and a cardiac rhythm supported by a pacemaker. He exhibited no neurological symptoms. During his stay in the Cardiothoracic Surgery Department, the patient had no further seizures despite the lack of any antiepileptic treatment.

During one year follow-up, the patient remained on a medication regimen that included oral warfarin, atorvastatin, zofenopril, torasemide, empagliflozin and pantoprazole. He remained seizure-free and fully independent, with no residual neurological symptoms and has regular check-ups in the cardiosurgery outpatient clinic.

**Figure 7:**
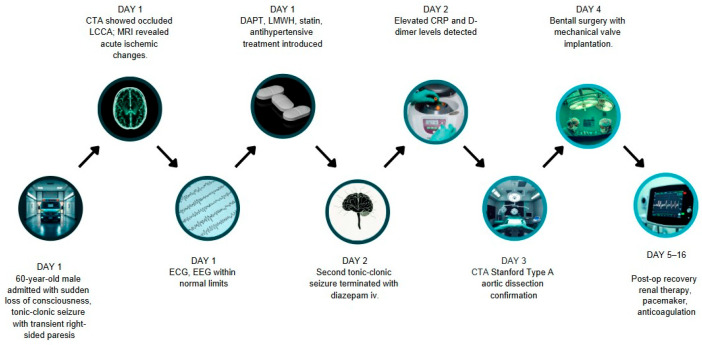
Timeline illustrates the sequence from admission to postoperative recovery period.

The timeline of the events is illustrated in Figure 7.

## 3. Discussion

In this case report, we describe a patient who suffered from a Type A Stanford/I DeBakey AoD which clinically primarily manifested as generalized tonic–clonic seizures.

Acute aortic dissection (AAD), alongside intramural hematoma (IMH), penetrating atherosclerotic ulcers (PAUs), and traumatic aortic dissection (TAD) are distinct conditions of acute aortic syndrome (AAS).

AASs are a diverse group of disorders which are typically symptomatic and share a set of signs and symptoms, the most prominent of which is chest discomfort. Although pain is a typical symptom, various other symptoms can be presented by the occlusive dissection of aortic branches, aneurysmal expansion of the dissected aorta, or hypotension related to hemopericardium [5].

Although AAS is rare, occurring in approximately 3.5 to 6.0 cases per 100,000 patient-years, rapid diagnosis is crucial as emergency surgical intervention is often necessary. According to a systematic review, chest or back pain was the most commonly reported symptoms of AAS, occurring in 61.6% to 84.8% of cases. Patients were typically aged between 60 and 70 years, with a predominance of males (50% to 81%), and many had hypertension (45% to 100%). The sensitivity of imaging techniques for diagnosing AAS is notable: computed tomography has a sensitivity of 100%, the sensitivity of magnetic resonance imaging ranges from 95% to 100%, and transesophageal echocardiography has a sensitivity of 86% to 100%. In contrast, blood D-dimer tests show a sensitivity range of 51.7% to 100% [6].

Various strategies have been proposed for AoD classification and two stand out, the DeBakey and Stanford classifications. DeBakey classifies aortic dissections into three types, depending on whether the ascending aorta, descending aorta, or both are involved. Stanford classification divides it into two main groups—type A if it involves the ascending aorta, and type B in cases where it does not [7,8]. The latter is widely used for simplicity and clear decision-making based on either type. Both scales separate AoD into those that need complete surgical replacement of the dissected portion and those that usually require only standard medical management.

Neurologic complications related to aortic dissection (AoD) are relatively untypical. In a retrospective study from Korea over 11 years, observation of type A and type B AoD revealed that 14.7% of patients had a neurologic complication, either an early-onset complication or a delayed-onset complication. The most frequent manifestation was ischemic stroke (9.4%), followed by hypoxic encephalopathy (9, 3.2%), ischemic neuropathy (5, 1.8%), spinal cord ischemia (5, 1.8%), seizure (2, 0.7%), hoarseness (1, 0.4%) and septic encephalopathy (1, 0.4%). The neurologically complicated group had a mortality rate of 43.9%. when overall in-hospital mortality was 10.1% [9].

In another study, neurological symptoms occurred in 26.0% of patients with AoD. The most common symptom was dizziness (6.5%), followed by syncope (5.7%), single lower-limb sensory disturbances (5.4%), single lower-extremity weakness (3.1%), coma (2.5%), paraplegia (2.2%), headache (1.5%) and hemiplegia (1.0%). It was also found that AoD may be first manifested by neurological symptoms, including syncope, dizziness and headache. Patients with type A aortic dissection were more vulnerable to the neurological symptoms than those with type B aortic dissection (34.6% vs. 14.7%) [10].

A study from Germany reported initial neurological symptoms in 29% of AoD patients. Only two-thirds of them reported chest pain, and most patients without initial neurological symptoms experienced pain (94%). Neurological symptoms were attributable to ischemic stroke (16%), spinal cord ischemia (1%), ischemic neuropathy (11%), and hypoxic encephalopathy (2%). Other frequent symptoms were syncopes (6%) and seizures (3%). In half of the patients, neurological symptoms were transient. Postoperatively, neurological symptoms were found in 48% of all patients, encompassing ischemic stroke (14%), spinal cord ischemia (4%), ischemic neuropathy (3%), hypoxic encephalopathy (8%), nerve compression (7%), and postoperative delirium (15%). Overall mortality was 23% and did not significantly differ between patients with and without initial neurological symptoms or complications [11].

A recent review of the neurological manifestations of AoD reports have revealed that 43.8% of patients displayed quantitative impairment of consciousness. Within this group, transient loss of consciousness was observed in 34.3% of cases. Motor syndrome was present in 54.5%, with 23.7% of patients presenting with hemiparesis or hemiplegia. A total of 3.6% had facial paresis or paralysis and less frequently monoparesis, paraparesis, hyperreflexia, spasticity, and muscle spasms. A total of 7.6% presented with language impairments, including Broca’s aphasia and dysarthria. Among patients diagnosed with Stanford type B, 57.0% displayed motor syndrome, with 50.0% displaying paraparesis or paraplegia, 4.7% displaying monoplegia, and 2.3% displaying hemiplegia or hemiparesis. Additionally, impairment of consciousness was present in 26.7%, sensory syndrome in 12.8%, and language impairments in 3.5%. Intriguingly, seizures were present in 3.6% of patients of AoD type A but were not seen in type B [12].

Another recent case report of a 54-year-old male described AoD presenting as an epileptic seizure, which was likely caused by cerebral hypoperfusion due to compromised blood flow from the dissection, whereas the patient developed hemodynamic instability, which prompted imaging beyond the neurological evaluation [13].

The occurrence of seizures in patients with AoD is believed to stem from reduced blood flow to the brain, potentially causing cerebral ischemia or hypoxia. Moreover, involvement of the carotid arteries in the dissection can lead to neurological deficits, including transient symptoms. Pathophysiologically, an acute type A aortic dissection can provoke seizures through several converging cerebrovascular mechanisms. Extension of the dissection into the carotid arteries—reported in about 30% of cases and strongly associated with neurological deficits—may compress the true lumen or create dynamic flow limitation, producing cerebral hypoperfusion [14,15]. This hemodynamic compromise can trigger diffuse or watershed ischemia, lowering cortical metabolic reserve and precipitating acute symptomatic seizures. In parallel, a thrombus within the false lumen or dissected carotid segment may embolize distally, generating focal cortical infarcts that further increase seizure risk. Both hemodynamic and embolic mechanisms often occur simultaneously in cerebral malperfusion syndromes. In the context of AoD, seizures are likely not the result of a primary epileptic disorder but rather consequences of both impaired cerebral perfusion and embolic cortical injury. Magnetic resonance imaging (MRI) of our patient revealed small, distally located foci without any signs of watershed infarcts, indicating that embolic processes and secondary focal ischemia were present at the time of the seizures. This also argued against recognizing symptoms observed in our patient as resulting from Todd’s paralysis, the well-recognized phenomenon characterized by a temporary, transient, post-ictal state of hemiplegia, weakness, or sensory loss occurring after an epileptic seizure [16]. Therefore, after complete resolution of all initial neurological deficits and reviewing ECG and EEG results, yet still confirming ischemic stroke in neuroimaging studies, we introduced the patient to dual antiplatelet therapy (DAPT). According to current guidelines, especially for patients with recent minor (NIHSS score ≤ 3) noncardioembolic ischemic stroke or high-risk transient ischemic attack (TIA), DAPT (aspirin plus clopidogrel) should be initiated early (ideally within 12–24 h of symptom onset and at least within 7 days of onset). Moreover, in patients with carotid artery stenosis and a TIA or stroke, intensive medical therapy with antiplatelet therapy, lipid-lowering therapy, and treatment of hypertension is recommended to reduce stroke risk [17]. Furthermore, a recent meta-analysis confirms that DAPT effectively reduces recurrent ischemic stroke and major adverse cardiovascular events (MACEs), especially when initiated within 12–24 h using aspirin plus clopidogrel [18].

However, when considering AoD therapy, it is crucial to avoid antithrombotics, especially thrombolytics, anticoagulants, and antiplatelet agents. These medications can lead to increased false lumen expansion, elevate the risk of aortic rupture, or delay life-saving surgical interventions. This is particularly important in cases where dissection may present symptoms similar to those of a stroke or myocardial infarction [19]. Although arterial dissection involves hemorrhage within the arterial wall, the primary cause of stroke is often artery-to-artery embolism resulting from an intraluminal thrombus. This could be the rationale for using antithrombotic agents to decrease the rate of ischemic stroke [20]. While DAPT is recommended for secondary stroke prevention, our patient’s case highlights the importance of clinical vigilance when deciding on such treatment. In this instance, it was only after the patient experienced a second seizure that further blood testing was conducted, which revealed an elevated D-dimer level. An additional angio-CT scan was performed, ultimately showing clear signs of dissection. At this point, DAPT was discontinued, and the patient was urgently referred to the cardiothoracic team. Moreover, LMWH is not an advisable treatment option for AoD. It is worth noting that while prophylactic doses of LMWH are recommended during the acute phase of a minor cardioembolic stroke, they are primarily intended to prevent venous thromboembolism or pulmonary embolism [21]. Furthermore, our patient was also promptly treated with antihypertensive drugs. Effective management of hypertension is vital in cases of aortic dissection [22]. According to a recent meta-analysis, among antihypertensive therapies, beta blockers were associated with the greatest reduction in MACEs, particularly in patients with type B dissection. Angiotensin-converting enzyme inhibitors (ACEis) and angiotensin receptor blockers (ARBs) were also associated with a significant reduction in MACEs [23]. However, it is important to exercise caution regarding the choice of agents [24]. Therefore, our case exemplifies not only an unusual clinical manifestation of AoD but also a clinical dilemma that underscores the need for a careful assessment of the benefits and risks associated with personalizing therapeutic approaches in patients experiencing acute seizures.

This case exemplifies an atypical neurological manifestation of AoD, specifically presenting as generalized tonic–clonic seizures. It emphasizes the importance of recognizing potential AoD in patients with such neurological symptoms. By maintaining vigilance for aortic pathology in these instances, healthcare professionals can facilitate timely diagnosis and management of this critical condition, ultimately improving patient outcomes.

## 4. Future Directions

We believe that implementing a proactive approach in future medical protocols should significantly enhance the chances of timely diagnosis and treatment of AoD, including atypical neurological presentations, leading to improved patient outcomes.

## 5. Conclusions

In summary, while seizures are an uncommon presentation of AoD, awareness of this association is essential for timely diagnosis and management, which can potentially improve patient outcomes. Given the possibility of atypical presentations, clinicians should maintain a high index of suspicion for AoD in patients presenting with unexplained seizures, especially when these are accompanied by chest pain, syncope, or signs of hemodynamic instability. Identification of symptoms such as hypotension, asymmetrical pulses, or heart murmurs, as well as thoroughly reviewing imaging studies in patients who do not have a history of epilepsy, should help to exclude an acute aortic pathology. Early recognition and intervention are crucial, as the mortality rate for untreated AoD rises significantly over time.

## Data Availability

No datasets were generated or analyzed during the current study.

## Abbreviations

ASPECTS	Alberta Stroke Program Early CT Score
AAS	Acute aortic syndrome
AAD	Acute aortic dissection
AoD	Aortic dissection
CT	Computed tomography
CTA	Computed tomography angiography
DAPT	Dual antiplatelet therapy
IMH	Intramural hematoma
LMWH	Low-molecular-weight heparin
MACEs	Major adverse cardiovascular events
mRS	Modified Rankin Scale
MRI	Magnetic resonance imaging
NIHSS	National Institutes of Health Stroke Scale
PAU	Penetrating atherosclerotic ulcer
TAD	Traumatic aortic dissection
TIA	Transient ischemic attack
